# Nebulised amphotericin B-polymethacrylic acid nanoparticle prophylaxis prevents invasive aspergillosis

**DOI:** 10.1016/j.nano.2015.02.012

**Published:** 2015-07

**Authors:** Khojasteh Shirkhani, Ian Teo, Darius Armstrong-James, Sunil Shaunak

**Affiliations:** Departments of Medicine, Infectious Diseases, Immunity and Chemistry, Imperial College London, Hammersmith campus, London, UK

**Keywords:** Amphotericin B, Polymethacrylic acid, Nebulised nanoparticle, *Aspergillus fumigatus*, TNF-α and Interferon-γ

## Abstract

*Aspergillus* species are the major life threatening fungal pathogens in transplant patients. Germination of inhaled fungal spores initiates infection, causes severe pneumonia, and has a mortality of > 50%. This is leading to the consideration of pre-exposure prophylaxis to prevent infection. We made a very low MWt amphotericin B-polymethacrylic acid nanoparticle. It was not toxic to lung epithelial cells or monocyte-derived-macrophages *in-vitro*, or in an *in-vivo* transplant immuno-suppression mouse model of life threatening invasive aspergillosis. Three days of nebuliser based prophylaxis delivered the nanoparticle effectively to lung and prevented both fungal growth and lung inflammation. Protection from disease was associated with > 99% killing of the *Aspergillus* and a 90% reduction in lung TNF-α; the primary driver of tissue destructive immuno-pathology. This study provides *in-vivo* proof-of-principle that very small and cost-effective nanoparticles can be made simply, and delivered safely and effectively to lung by the aerosol route to prevent fungal infections.

**From the Clinical Editor:**

Aspergillus is an opportunistic pathogen, which affects immunocompromised patients. One novel way to help fight against this infection is pre-exposure prophylaxis. The authors here made PMA based anionic hydrogels carrying amphotericin B, with mucoadhesive behavior. They showed that aerosol route of the drug was very effective in protecting against the disease in an in-vivo model and should provide a stepping-stone towards clinical trials in the future.

## Graphical abstract

Nebulised nanoparticle prevents invasive aspergillosis. *Aspergillus* species are the major life threatening fungal pathogens in transplant patients. They cause severe pneumonia with mortality of > 50%. Preventing infection is therefore being explored. This very low MWt amphotericin B-polymethacrylic acid nanoparticle was given as an aerosol to a transplant immuno-suppression mouse model with life threatening invasive aspergillosis. Protection was associated with > 99% killing of the *Aspergillus* and a 90% reduction in lung TNF-α related inflammation. Simple and cost-effective pre-exposure prophylaxis prevents invasive lung fungal infections.

**Figure:**
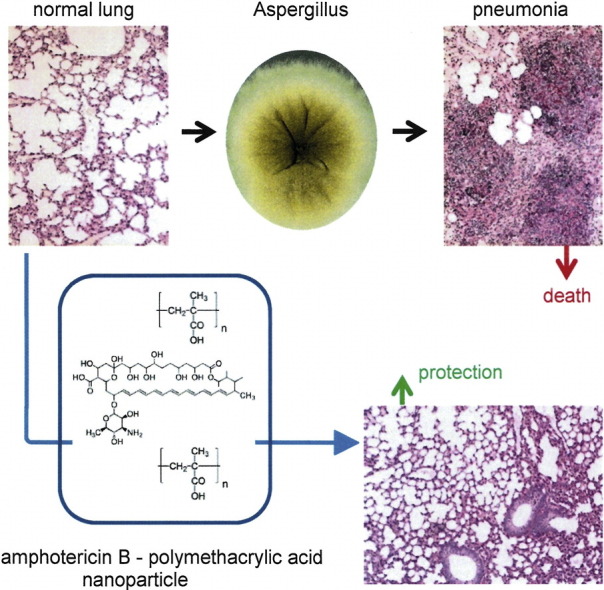

Nanoparticles made from functional polymers using synthetic chemistry are a rapidly growing area of interest in nanomedicine.^1–3^ Self-assembled hydrogels are also being developed to encapsulate hydrophobic drugs for sustained drug delivery.^4^ These materials are being investigated and applied clinically for direct delivery of drugs to sites of disease.^5^

We have previously shown that established, scalable and low-cost synthetic chemistries can be used to make a water soluble amphotericin B-PMA (AmB-PMA) using an 18.5 kDa polymethacrylic acid-Na. It was safe and subcutaneous injection led to rapid cure of cutaneous leishmaniasis in a non-healing disease mouse model.^6^ PMA based anionic hydrogels also exhibit mucoadhesive behaviour that enables continuous release of active drug for > 8 h in the respiratory tract.^7^ This has led us to define the smallest possible PMA-Na for making an AmB-PMA nanoparticle for aerosol delivery to the lung. Our aim was to provide a simple, low toxicity, and highly effective means of pre-exposure prophylaxis against invasive aspergillosis in immuno-suppressed patients; e.g. solid organ transplants, hematopoietic stem cell transplants, AIDS, cancer. This is because patient mortality from invasive aspergillosis remains > 50% despite improvements in immuno-suppressive drug regimens and new antifungal drugs.^8,9^ Prevention rather than cure is now becoming the preferred clinical option.

Respiratory inhalation of environment spores is responsible for 90% of all cases of invasive aspergillosis.^10–12^ Germination of spores in small bronchioles initiates infection, causes severe pneumonia, and has a mortality of > 50%.^13^ This led us to consider pre-exposure prophylaxis to prevent infection with as few inhaled doses as possible, and without pulmonary toxicity.

## Methods

### Synthesis and characterisation

These fully hydrolysed analytical standard grade polymethacrylic acid sodium salts were used: 18,500 Da PMA; Mp 18,500, Mw 18,600, (Sigma-Aldrich 02356) and 7750 Da PMA; Mp 7830, Mw 7750 (Sigma-Aldrich 02355) and 3520 Da PMA; Mp 3480, Mw 3520 (Polymer Standards Services Mainz [PSSM] PSS-pma 3.5k) and 1700 Da PMA; Mp 1670, Mw 1700 (PSSM PSS-pma1.6k) and 1270 Da PMA; Mp 1250, Mw 1270 (Sigma-Aldrich 02351).

The following aspects of the synthesis previously described^6^ were further simplified and optimised. 50 mg PMA-Na was dissolved in 10 ml water and solubilised on a rotary shaker at 20°C overnight. 9.7 ml DMSO was added to 50 mg AmB, sealed under argon, and stirred for 3 h. 10 ml of 5 mg/ml PMA-Na solution was treated twice with activated charcoal (12.5 mg, 25% w/w) to remove endotoxin. After each treatment, the charcoal-PMA slurry was centrifuged at 13,000 × *g* for 8 min. The supernatant was removed and filtered twice through a 0.2 μm filter to remove non-solubilised particles. The AmB solution (2 ml) was added to a round-bottom glass flask and 1 N NaOH (100 μl, 15 μl/3 s) added dropwise, followed by the addition of the PMA-Na solution (2 ml) and 8 ml water, and stirred for 1 h at 20 °C. It was then transferred to a Float-a-Lyzer (500-1000 Da cut-off) or Slide-a-Lyzer cassette (2-3.5 kDa cut-off depending upon PMA-Na size) and dialysed against sterile water for 4 h at 4 °C. Water was changed and the dialysis continued for 20 h at 4 °C; during this time it was important to regularly adjust the pH of the AmB-PMA dialysate to 12. The dialysed solution was lyophilised to give a pale yellow powder. The amount of AmB in AmB-PMA was determined by UV spectroscopy as described.^6^

### *In-vitro* biology

Anti-fungal activity against *A. fumigatus* was determined using the Clinical & Laboratory Standards Institute (CLSI) method.^14^ *A. fumigatus* strain CEA10 conidia were harvested from an SDA plate in 0.1% Tween 20 and filtered through Miracloth (Millipore). Conidia were re-suspended in sterile saline, counted, and adjusted to 10^4^-10^5^ conidia/ml. Doubling dilutions of the AmB-PMA nanoparticles in 100 μl of RPMI 1640, 2% glucose, 0.165 mol/l MOPS pH 7.0 (range 0.0156-16 μg/ml) were dispensed into 96-well plates and inoculated with 100 μl of conidia (10^4^-10^5^ conidia/ml) per well in accordance with the CLSI M38a protocol. Plates were incubated in a sealed container for 48 h at 37 °C and then assayed for fungal growth by microscopy. The minimum inhibitory concentration (MIC) was defined as the concentration beyond which no fungal growth occurred.

### Fungal burden in mouse lung

The first method used was colony forming unit (CFU) counting. Lung tissue was mechanically disrupted in 1 ml sterile saline and the fungal spores and hyphae released serially diluted and spread on SDA plates. They were then incubated for 24 h at 37 °C and the number of *A. fumigatus* colonies counted. The second method used quantitative PCR. Tissue was homogenised in 250 μl PBS and DNA extracted using a DNeasy Blood and Tissue kit (Qiagen). 180 μl of buffer ATL and 20 μl proteinase K solution were added to 250 μl homogenate lung and incubated overnight at 56 °C. These samples were then processed according to the manufacturer’s instructions and applied to DNeasy columns, and the DNA diluted in 200 μl elution buffer.

The ADR1 (CAGGCTGGCCGCATTG) and ASF1 (GCACGTGAAATTGTTGAAAGG) primer pairs were used to amplify the fungal 28S rRNA gene with amplified product detection based upon hybridisation to the ASP28P TaqMan fluorescent probe [6 FAM] CATTCGTGCCGGTGTACTTCCCCG [TAM]. The fungal burden was quantitated by PCR and normalised against murine β-actin using the forward primer CGAGCACAGCTTCTTTGCAG and the reverse primer CCCATGGTGTCCGTTCTGA as described.^15^

### Mouse lung cytokine assays

Quantitative RT-PCR of mRNA in mouse lung was used for the analysis of TNF-α, IFN-γ, MIP-1β, IL-10 and iNOS as described.^6,9,16^

### *In-vivo* efficacy

BALB/c and C57BL/6 mouse models of life threatening invasive aspergillosis in solid organ transplantation were used as described.^17,18^ In brief, mice were immuno-suppressed with 125 mg/kg hydrocortisone given subcutaneously at day − 3 and day − 1 before infection (which was defined as day 0), and again at day + 2 and + 5 after infection. In addition, FK-506 was given intraperitoneally at 1 mg/kg once daily from day − 3 onwards.

On the day of infection, mice were given isoflourane anaesthesia and 5 × 10^6^ *A. fumigatus* CEA10 spores in 40 μl normal saline inoculated intra-nasally; control mice received normal saline only. They were culled on days + 1, + 2, + 4 and + 8; control mice were culled on day + 2 because they had lost 20% body weight. This new mouse model of invasive aspergillosis in organ transplant patients is the first pathologically accurate representation of modern immuno-suppressive drug regimens which use a combination of a steroid (hydrocortisone) with a calcineurin inhibitor (FK-506; tacrolimus).^17,18^

A Perspex chamber that housed 4 mice was constructed using the method of Manunta et al.^19^ The clinically approved *AeroEclipse II* nebuliser, a disposable cost-effective and high output efficiency nebuliser, was used for nebulising the AmB-PMA nanoparticle after reconstitution in sterile water to give a yellow solution. The nebuliser was operated by an air cylinder at 3 bar pressure with 8 l/min airflow as is conventional for nebulisation protocols in patients. The total aerosolised dose of AmB in AmB-PMA dispensed into the chamber ranged from 40 to 400 μg/nebulisation. It was given on days − 3, − 2 and − 1 before infection with *A. fumigatus* (day 0) and was based upon previous industry animal model protocols.^20^ Control mice received nebulised water only or nebulised PMA-Na dissolved in water. After infection with *A. fumigatus*, hydrocortisone and FK-506 were continued until the mice were culled. No AmB-PMA was given after infection.

### *Ex-vivo* efficacy of nanoparticle

This was determined by nebulising different doses of the AmB-PMA with a Sabouraud dextrose agar plate placed inside the perspex chamber. *A. fumigatus* (1 × 10^7^) was then added to the plate and incubated for 24 h at 37 °C.

### Quantitation of lung inflammation

Formalin fixed sections of lung tissue were cut and stained with PAS. ImageJ is a public domain Java image processing program (http://rsbweb.nih.gov/ij/) that can be used to calculate the area and pixel value statistics of user-defined selections. It was used to quantitate lung inflammation in tissue sections^17,18^ (n = 3).

### Data analysis

Graphpad Prism software was used. Results are shown as the mean ± SEM. *P* values were determined using a Mann–Whitney test.

## Results

### PMA-Na polymers

No haemolysis occurred with any of the analytical standard grade PMA-Na polymers used when they were incubated with fresh human red blood cells for 1 h up to 2.5 mg/ml. In an MTT assay, these PMA-Na polymers were not toxic to primary human monocyte-derived-macrophages or to lung epithelium A549 cells up to 500 μg/ml, and they had no immuno-modulatory activity (Supplementary Figure 1). In addition, they had no antifungal activity when tested against 11 isolates of *A*. *fumigatus* up to 256 μg/ml. These results showed that there were no biological characteristics that could distinguish between the various PMA-Na polymers used.

### AmB-PMA characterisation with varying weight PMA-Na

No differences were seen by UV spectrophotometry between the various AmB-PMAs synthesised and stored as pale yellow powders using PMA-Na with a MWt range of 1.27-18.5 kDa. The sustained alkalinisation of the AmB-PMA nanoparticle at pH 12 during dialysis was the crucial determinant of the peak UV fluorescence shifting from 329 nm, as seen for deoxycholate-AmB (Fungizone, Squibb), to 324 nm as seen for liposomal AmB (Ambisome, Gilead) (Supplementary Figure 2).^6,21^ All nanoparticles were confirmed to be endotoxin free using a limulus amoebocyte assay (i.e. endotoxin < 0.06 EU/ml; EU standard for water for injection) prior to biological testing.

When the antifungal activity of the AmB-PMA nanoparticles was determined by MIC testing using 11 isolates of *A. fumigatus* with deoxycholate-AmB as the positive control, their mean MICs ranged from 0.75 μg/ml for AmB-PMA (18.5 kDa) to 0.125 μg/ml for AmB-PMA (3.52 kDa) (n = 3). This compared to 0.25 μg/ml for deoxycholate-AmB (Supplementary Table 1). These results showed that the AmB-PMA (3.52 kDa) {AP}, whose AmB loading was 18% (compared to an AmB loading of 45% in deoxycholate-AmB (Fungizone, Squibb) and an AmB loading of 12.5% in liposomal AmB (Ambisome, Gilead)), was the least toxic nanoparticle to monocyte-derived-macrophages and also had the greatest antifungal activity against *A. fumigatus*. This made AP the best candidate for further *in-vitro* and *in-vivo* testing.

### Detailed *in-vitro* characterisation of AP

**Figure 1:**
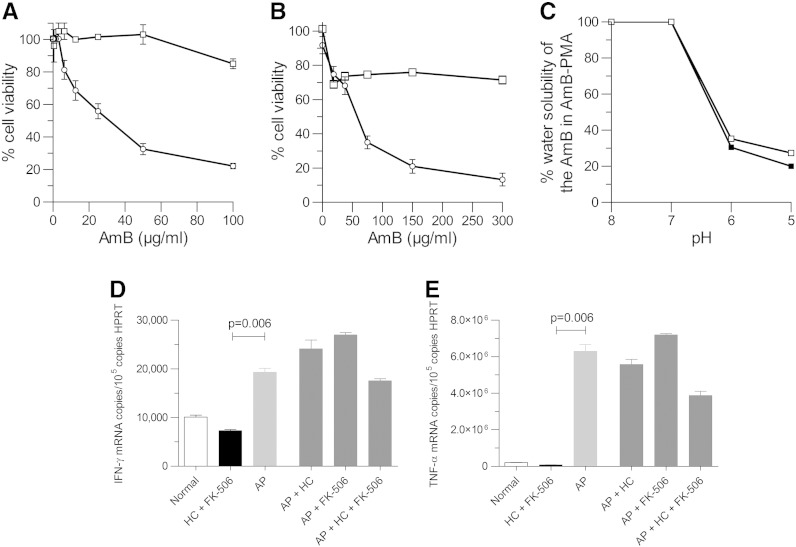
*In-vitro* studies of AmB-PMA (3.52 kDa) [AP]: (**A, B**) Viability of A549 cells **(A)** and MDMs **(B)** with AmB-PMA (3.52 kDa) [AP] & dexoycholate-AmB. 10^6^ cells were incubated with AmB-PMA (□) or dexoycholate-AmB (○) for 3 h and cell viability determined by MTT. No toxicity of AP was seen in epithelial A549 cells (n = 5) or monocyte-derived-macrophages (n = 25). **(C)** pH related solubility of AP in water. More free bioactive AmB was released from the AmB-PMA (3.52 kDa) (□) at pH 5 (i.e. 28%) than from the AmB-PMA (18.5 kDa) (■) (i.e. 20%) (*P* < 0.01; n = 3). **(D)** The IFN-γ and TNF-α immuno-stimulatory activity of AP on monocyte-derived-macrophages was not altered by the presence of therapeutically relevant doses of hydrocortisone and FK-506. For IFN-γ: Normal = 10,080 ± 410 copies/10^5^ copies HPRT; HC + FK-506 = 7280 ± 270 copies/10^5^ copies HPRT; AP = 19,280 ± 840 copies/10^5^ copies HPRT. For TNF-α: Normal = 203,600 ± 3500 copies/10^5^ copies HPRT; HC + FK-506 = 79,300 ± 940 copies/10^5^ copies HPRT; AP = 6.29 × 10^6^ ± 338,000 copies/10^5^ copies HPRT.

AP was not toxic to either lung epithelium A549 cells (Figure 1, *A*) or monocyte-derived-macrophages (Figure 1, *B*). Changing the PMA-Na from MWt 18.5 kDa to 3.52 kDa changed the diameter of the AmB-PMA nanoparticle from 94 ± 1 nm to 78 ± 9 nm respectively as measured by Dynamic Light Scattering. This compares to a diameter of 75 nm for deoxycholate-AmB and 78 nm for liposomal-AmB. Nanoparticles of this size are effectively delivered to the lung’s terminal bronchioles.^22,23^

At an alkaline pH, the carboxylic acid groups of PMA are ionised and repel each other; this allows the PMA hydrogel to swell and trap small molecules like AmB within its matrix. At an acidic pH, the carboxylic acid groups are de-ionised and the hydrogel collapses; this results in the AmB being released into solution.^6^ We found that more AmB was released from AP (i.e. 3.52 kDa) into solution at pH 5 than from AmB-PMA (18.5 kDa); 28% versus 20% respectively (*P* < 0.01) (Figure 1, *C*).

The *in-vitro* immuno-modulatory activity of AP on monocyte-derived-macrophages was also evaluated in the presence of therapeutically relevant doses of hydrocortisone and FK-506 by measuring the cytokines TNF-α & IFN-γ. There was no significant effect of hydrocortisone or FK-506, or of the combination of hydrocortisone and FK-506, on IFN-γ or TNF-α. In contrast, the addition of AP significantly increased both IFN-γ (Figure 1, *D*) and TNF-α (Figure 1, *E*).

### *A. fumigatus* infection in BALB/c and C57BL/6 mouse species

**Figure 2:**
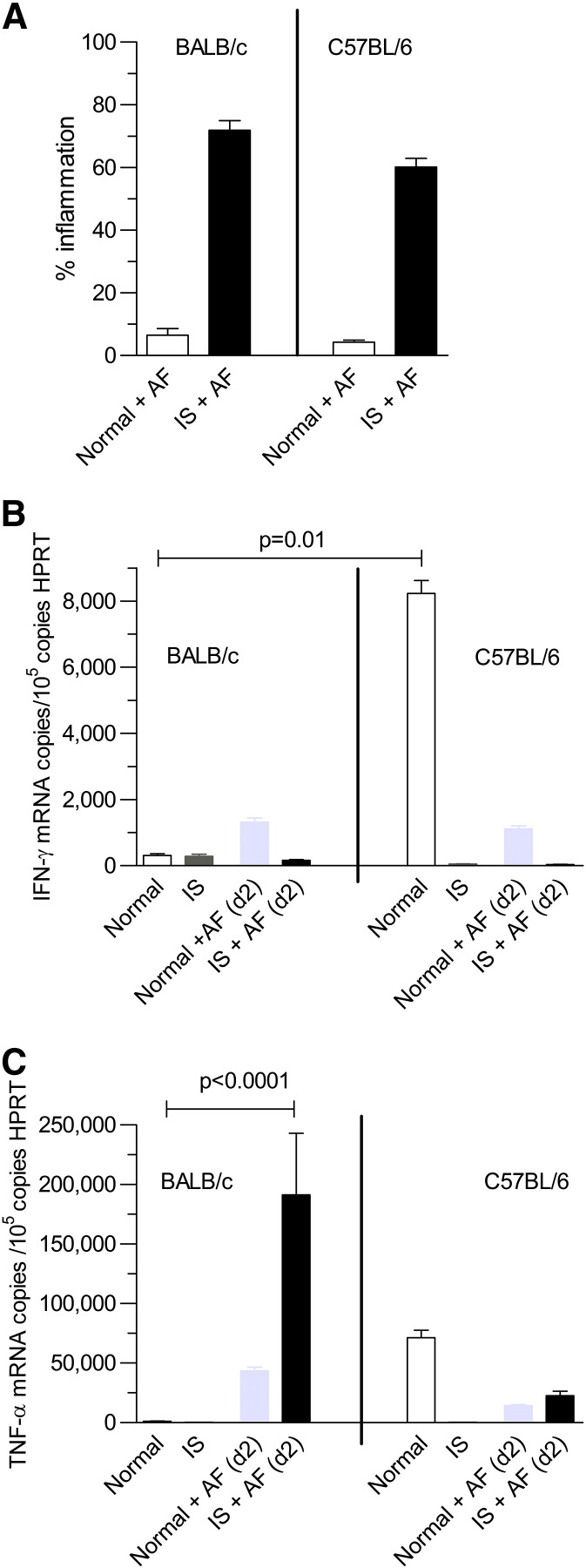
Comparison of BALB/c and C57BL/6 mouse lung: **(A)** Percentage inflammation in lung was similar in BALB/c and C57BL/6 mice (n = 3). **(B)** IFN-γ in normal (i.e. immuno-competent) C57BL/6 mouse lung was much higher than in normal BALB/c mouse lung. IFN-γ was depressed in immuno-suppressed (IS) BALB/c and C57BL/6 mouse lung on day + 2 after infection (n = 4). Normal IFN-γ for BALB/c mouse = 310 ± 56 copies/10^5^ copies HPRT; Normal IFN-γ for C57BL/6 mouse = 8230 ± 395 copies/10^5^ copies HPRT. **(C)** There was a large increase in TNF-α in immuno-suppressed (IS) BALB/c mouse lung at day + 2 after infection (*P* < 0.0001) (n = 4). Normal TNF-α for BALB/c mouse = 1230 ± 120 copies/10^5^ copies HPRT; IS + AF (d + 2) TNF-α for BALB/c mouse = 191,300 ± 51,700 copies/10^5^ copies HPRT. Normal TNF-α for C57BL/6 mouse = 191,300 ± 51,700 copies/10^5^ copies HPRT; IS + AF (d + 2) TNF-α for C57/BL mouse = 22,700 ± 3600 copies/10^5^ copies HPRT.

There was minimal inflammation when normal BALB/c and C57BL/6 mice were intra-nasally challenged with *A. fumigatus* (0-6.5 ± 1.2% (n = 10)). When immuno-suppressed mice were infected, there was a large increase in lung inflammation at day + 2 which was similar in BALB/c and C57BL/6 mice (60-74 ± 3% (n = 10)) (Figure 2, *A*). Although IFN-γ was similar in both species after infection (Figure 2, *B*), TNF-α was much higher in BALB/c mice (Figure 2, *C*). As a high level of TNF-α is known to be the primary driver of the destructive immuno-pathology associated with invasive aspergillosis in the lungs of immuno-suppressed patients,^9,17,18^ this suggested that the BALB/c mouse model of invasive aspergillosis was the most appropriate for undertaking further evaluation of the *in-vivo* bioactivity of pre-exposure prophylaxis with AP.

### *In-vitro* efficacy of the AeroEclipse II in nebulising AP

The AeroEclipse II nebuliser was used to nebulise the nanoparticle in the dose range 40-400 μg AmB in AP. Over a 30 min period, the aerosol spread evenly throughout the perspex box and onto the agar plate in its bottom. The plate was then inoculated with *A. fumigatus* and cultured for 24 h. Many apergillus colonies grew at 40 μg of AmB in AP, and some colonies still grew at 200 and 250 μg of AmB in AP. In contrast, no fungal growth occurred with 300 μg of AmB in AP.

### AP pre-exposure prophylaxis in BALB/c mice at day + 2

The immuno-suppressed and infected mice were culled at day + 2 after infection because of loss of 20% body weight. At a dose of 40 μg AmB in AP, there was no prophylactic benefit seen using any of the following parameters; histology, CFU, quantitative PCR for *Aspergillus* ribosomal 28S rRNA, and quantitative RT-PCR of mRNA for analysis of TNF-α, IFN-γ, MIP-1β, IL-10 and iNOS in mouse lung (n = 6/group).

**Figure 3:**
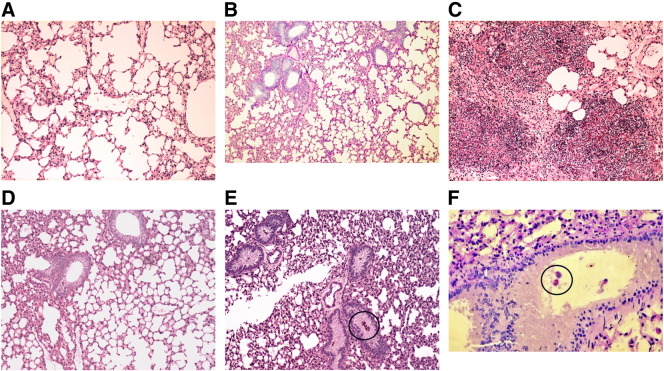
PAS stain histology of lung tissue: **(A)** Normal BALB/c mouse lung (× 10). **(B)** Normal BALB/c mouse lung at day + 2 after being exposed to *A. fumigatus* (× 10). The % inflammation in the tissue sections was 6.5 ± 2.1% (n = 4). **(C)** Immuno-suppressed and *A. fumigatus* infected BALB/c mouse lung at day + 2 (× 10). The % inflammation in the tissue sections was 74 ± 3% (n = 6). **(D)** Immuno-suppressed BALB/c mouse lung with AP pre-exposure prophylaxis of 300 μg AmB in AP for 3 days and *A. fumigatus* infection at day + 2 (× 10). The % inflammation in the tissue sections was 14 ± 3% (n = 6). **(E, F)** Immuno-suppressed BALB/c mouse lung with AP prophylaxis and *A. fumigatus* infection at day + 2 showing spores in terminal bronchioles **(E**; × 10**)** and in alveolar macrophages **(F**; × 40**)**. No invasive fungal hyphae were seen. *A. fumigatus* were occasionally seen as spores engulfed by alveolar macrophages; these have been highlighted within the black circles.

With a dose of 200 or 250 μg AmB in AP, the mice did not lose weight (i.e. < 5%), and there was a large reduction in fungal growth when compared to the immuno-suppressed and infected controls. However, fungal growth from the lungs at day + 2 was not eliminated by this dose; this only occurred when a dose of 300 μg AmB in AP was used (Figure 3). No further benefit was seen by increasing the dose to 400 μg AmB in AP. Therefore, the additional studies were performed with a once daily dose of 300 μg AmB in AP given for 3 consecutive days before infection.

**Figure 4:**
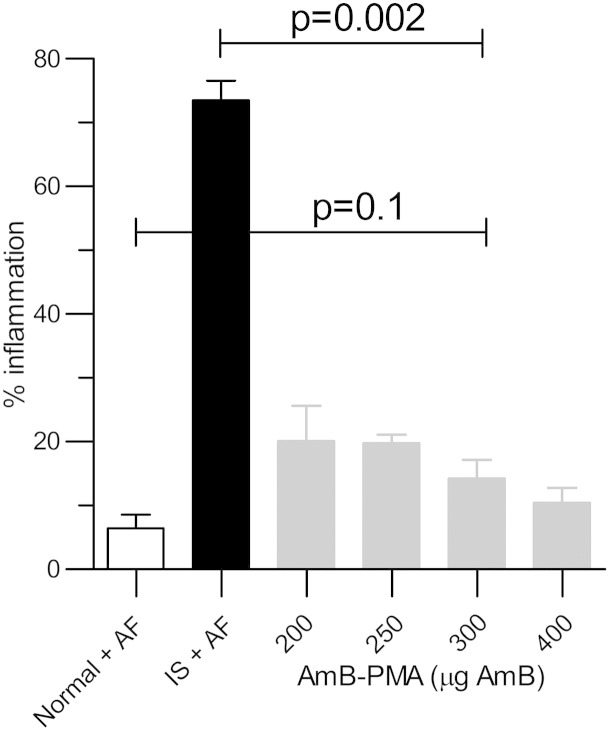
Percentage inflammation in the tissue sections from BALB/c mouse lung after prophylactic AP. There was a significant reduction in lung inflammation (*P* = 0.002) after pre-exposure prophylaxis with AP at a dose of 300 μg AmB given for 3 consecutive days. The degree of inflammation seen (14 ± 3%) was similar to that seen in normal mice exposed to *A. fumigatus* (*P* = 0.1; n = 4).

At autopsy, the most striking difference was that lungs from both normal and immuno-suppressed/infected/AP pre-exposure prophylaxis mice had a normal macroscopic appearance when compared to the blackened lungs of the immuno-suppressed/infected mice. The percentage inflammation in BALB/c lung with a dose of 300 μg AmB in AP (14 ± 2.3% (n = 6/group)) was similar to that seen in normal BALB/c mice exposed to aspergillus spores (6.5 ± 2.1%; *P* = 0.1 (n = 4/group)). This was considerably less than the inflammation seen in immuno-suppressed/infected mice (74 ± 3%; *P* = 0.002 (n = 6)) (Figure 4).

**Figure 5:**
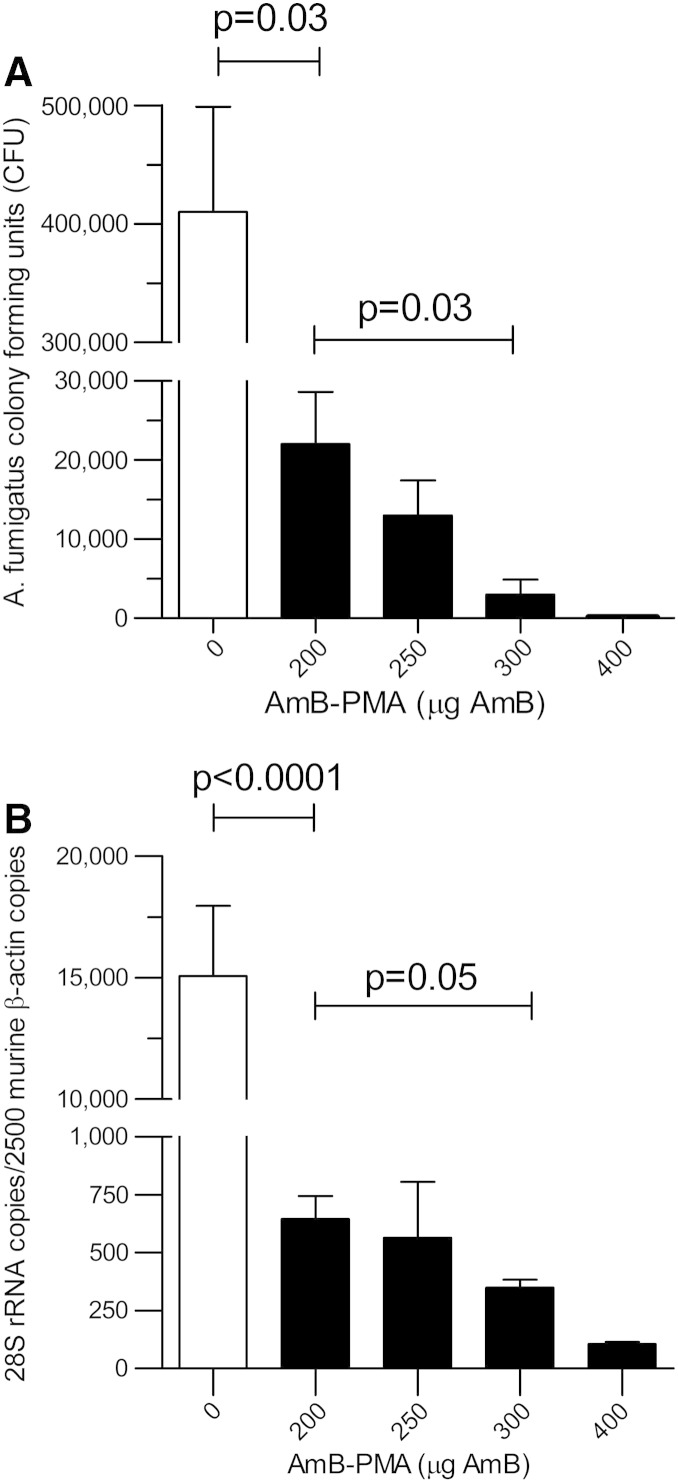
Fungal burden in immuno-suppressed BALB/c mouse lung after pre-exposure prophylaxis with AP at day + 2 post infection: **(A)** CFUs after plating on agar (n = 4). At 0 μg AmB, the CFUs were 410,000 ± 88,600; at 200 μg AmB, the CFUs were 22,000 ± 6600, and at 300 μg AmB, the CFUs were 3000 ± 1920 (a reduction of 99.3%). **(B)** As quantified by quantitative PCR for the 28S rRNA gene (n = 4). At 0 μg AmB, the 28S rRNA copies/2500 murine β-actin copies were 15,070 ± 2900; at 200 μg AmB, they were 646 ± 99, and at 300 μg AmB, they were 349 ± 34 (a reduction of 86%).

**Figure 6:**
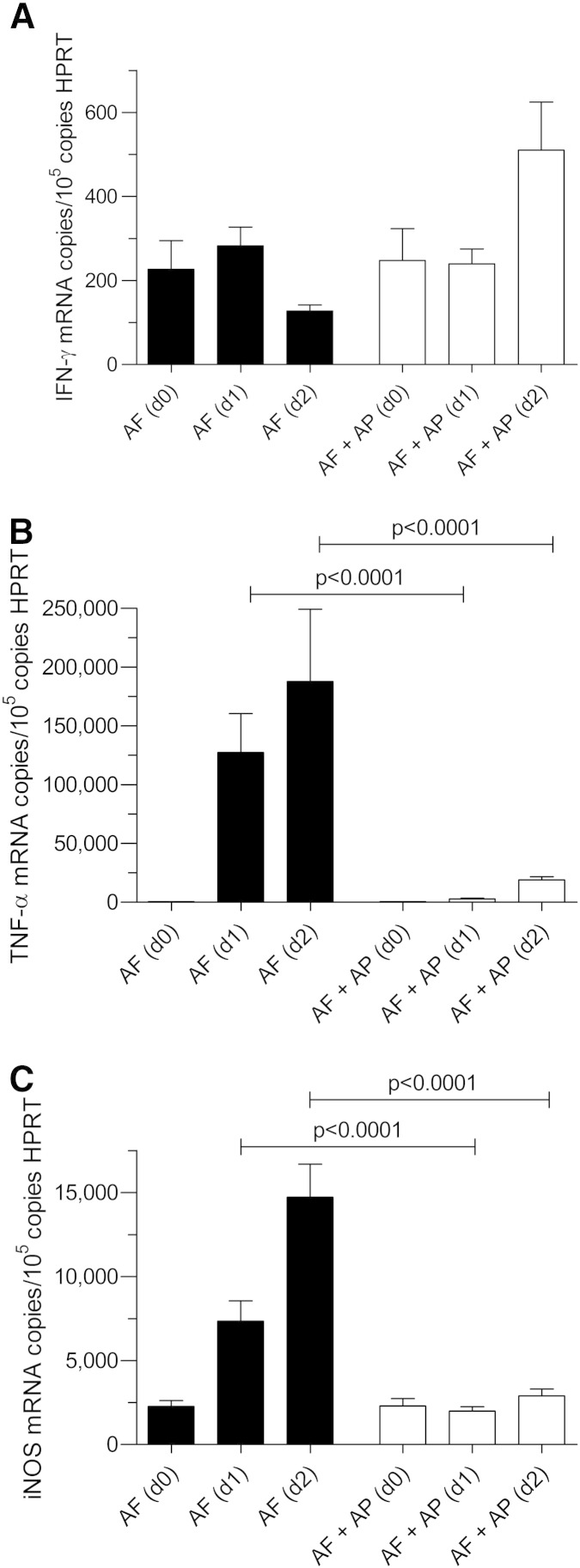
Cytokines in immuno-suppressed BALB/c mouse lung after pre-exposure prophylaxis with AP and day + 2 after infection: **(A)** For IFN-γ, no differences were seen. **(B)** TNF-α rose rapidly in immuno-suppressed and infected mice over the course of the first 2 days after infection. This time dependant increase was reduced by 90% in mice given AP pre-exposure prophylaxis (*P* < 0.0001; n = 4). AF (d0) = 512 ± 41 copies/10^5^ copies HPRT; AF (d + 2) = 187,800 ± 61,500 copies/10^5^ copies HPRT; AF + AP (d0) = 531 ± 86 copies/10^5^ copies HPRT; AF + AP (d + 1) = 2880 ± 596 copies/10^5^ copies HPRT; AF + AP (d + 2) = 19,085  ±  2370 copies/10^5^ copies HPRT. **(C)** iNOS did not increase in mice given AP prophylaxis suggesting that lung alveolar macrophage activation did not occur (n = 4). When compared to the iNOS in immuno-suppressed/infected mice at day + 2, iNOS was reduced by 90%.

The lung fungal burden at day + 2 after infection showed a large reduction in the pre-exposure prophylaxis AP group as measured by both CFU and 28S rRNA analysis (*P* < 0.0001) (n = 4) (Figure 5). Although there was no associated change in IFN-γ (Figure 6, *A*), there was, in contrast, a large and sustained reduction of 90% in TNF-α in the pre-exposure prophylaxis AP group (*P* < 0.0001) (n = 4) (Figure 6, *B*) that was also reflected in an 80% reduction in lung iNOS (*P* < 0.0001) (n = 4) (Figure 6, *C*). This suggested that lung alveolar macrophage activation did not occur when immuno-suppressed mice were given pre-exposure prophylaxis with AP.

### AP pre-exposure prophylaxis in BALB/c mice at day + 8

**Figure 7:**
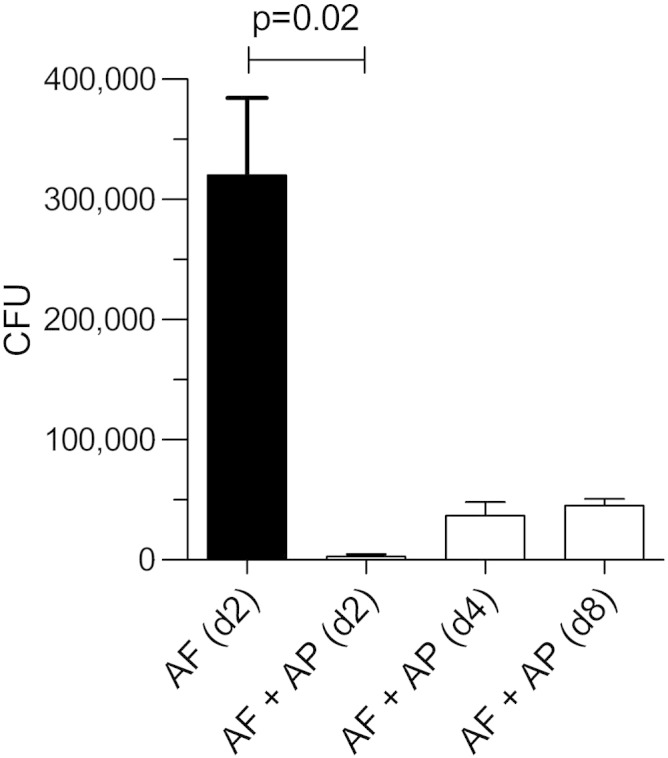
Fungal burden in immuno-suppressed BALB/c mouse lung after pre-exposure prophylaxis with AP at day + 8 post infection. At day + 8 after infection, there was an increase in CFUs as compared to the CFUs on day + 2 after infection (n = 4 mice/time point). However, this was 86% less that the CFU count in mice on day + 2 that had not received PA. For AF (d + 2), the CFUs were 320,000 ± 64,400; for AF + AP (d + 2) the CFUs were 2800 ± 1880, and for AF + AP (d + 8) the CFUs were 45,200 ± 5760.

**Figure 8:**
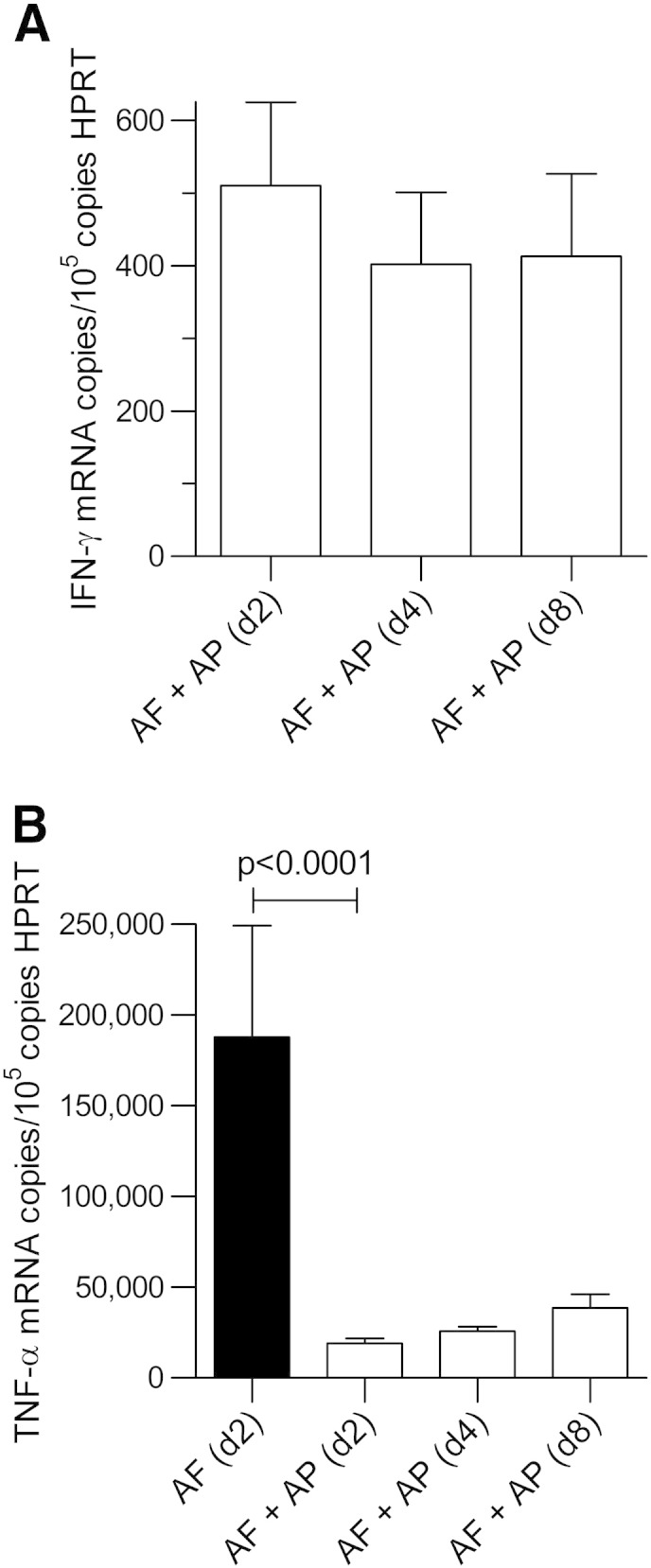
Cytokines in immunosuppressed BALB/c mouse lung after pre-exposure prophylaxis with AP at day + 8 after infection: **(A)** For IFN-γ, no differences were seen. **(B)** TNF-α at day + 8 showed a small increase when compared to day + 2 after infection in mice with AP pre-exposure prophylaxis (n = 4 mice/time point). AF (d + 2) = 187,800 ± 61,500 copies/10^5^ copies HPRT; AF + AP (d + 2) = 66,270 ± 2730 copies/10^5^ copies HPRT; AF + AP (d + 8) = 80,900 ± 7550 copies/10^5^ copies HPRT.

The immuno-suppressed and infected mice were culled by day + 2 because of 20% weight loss. Their fungal burden at that time was 320,000 ± 64,000 CFU/mouse; this figure was defined as 100%. In contrast, the fungal burden in the immuno-suppressed mice at day + 2, which had received pre-exposure prophylaxis with AP was 0.88 ± 0.59%; i.e. a > 99% reduction of the inoculum of 5 × 10^6^ *A. fumigatus* CEA10 spores administered had been eliminated in the first 48 h after infection by AP pre-exposure prophylaxis. Furthermore, by day + 8, these mice had lost only 10% of their body weight (n = 4), and the fungal burden in their lungs was 14.1 ± 1.8% of that in mice that had not received AP (Figure 7). There was no change in IFN-γ during the course of this experiment (Figure 8, *A*). In contrast, TNF-α was only 10 ± 1.43% of that in immuno-suppressed/infected mice at day + 2 (i.e. when these control mice were culled). It rose to 20 ± 3.9% (*P* < 0.0001) (n = 4) by day 8 (Figure 8, *B*).

## Discussion

Using a very small analytical standard grade polymethacrylic acid^24–26^ and simple, optimised, established, scalable and low cost synthetic chemistries, we have succeeded in turning insoluble and toxic AmB into a 3.52 kDa water soluble AmB-PMA nanoparticle of diameter 78 ± 9 nm. Its UV spectral analysis was made identical to liposomal AmB by ensuring that dialysis was performed at pH 12 (Supplementary Figure 2).^6,21^ The nanoparticle was soluble in water at 4 mg/ml and both stable and bioactive after storage at 4 °C for 18 months.

In a new solid organ transplantation mouse model of invasive aspergillosis that is representative of clinical practice today,^17,18^ three prophylactic doses of nebulised AP were sufficient to protect BALB/c and C57BL/6 mice from the life threatening pneumonia of invasive aspergillosis. The previous model of Schmitt et al^27^ was developed in 1988 and used hydrocortisone only as the immuno-suppressant. Our results suggest that 3 days of pre-exposure prophylaxis could be sufficient to protect immuno-suppressed patients from a range of life threatening and invasive fungal infections of the lung given the broad spectrum anti-fungal activity of AmB. The pre-exposure prophylaxis approach described for preventing fungal infections has both considerable clinical utility and cost saving potential. By comparison, current approaches to invasive aspergillosis are based upon tens of thousands of dollars for treating the pneumonia after it has become established. Mortality remains unchanged at > 50% despite the use of the latest anti-fungal drugs, and resistance to *Aspergillus* is already emerging. The aerosol administration of an AmB based nanoparticle therefore offers a targeted, cost-effective and patient friendly approach which could be combined with a mini portable nebuliser for home use. This compares very favourably with the daily intravenous administration of AmB for several weeks in hospital to treat established disease.

There is no vaccine for *Aspergillus*. Host protective immunity depends upon a highly coordinated interaction between innate and adaptive immunity. In normal mice exposed to *A. fumigatus*, a few inflammatory infiltrates containing neutrophils and monocytes are seen in the peribronchial region (Figure 3, *B*). This is because hyphae are rapidly killed by resident alveolar macrophages and incoming neutrophils; spores however can only be killed by resident and activated alveolar macrophages.^28^ This contrasts with the findings in hydrocortisone immuno-suppressed mice where numerous septate branching hyphae penetrate into lung tissue to cause bronchial wall damage and peribronchial necrosis (Figure 3, *C*).

Although some macromolecular polymers have immuno-modulatory properties,^29–31^ we have reported that this is not the case for 18.5 kDa PMA.^6^ We now show that this is also not the case for any of the PMA polymers with MWts from 1.27 to 18.5 kDa. However, the pH dependant release of AmB from the 3.52 kDa AP nanoparticle was 40% greater at pH 5 than it was from the 18.5 kDa AmB-PMA (Figure 1, *C*). This means that more of the free and bioactive AmB was present in high concentration in the macrophage endosome in which *Aspergillus* survives and persists.

In immuno-competent mice, the 18.5 kDa AmB-PMA killed leishmania amastigotes and the PMA in the macrophage endosome enhanced the host’s T-helper type 1 (Th1) cytokine response by stimulating IFN-γ production.^6^ This cytokine enhanced parasite killing. In contrast, in this study of invasive aspergillosis, hydrocortisone induced immuno-suppression led to a marked suppression of Interferon Regulatory Factor (IRF)-1 (Supplementary Figure 3).^32–34^ This disabled the IFN-γ mediated response in mouse lung to invasive aspergillosis. Previous studies have shown the importance of IFN-γ in curing aspergillosis: (i) In the steroid & cyclophosphamide based mouse model of invasive aspergillosis, death was primarily associated with high levels of TNF-α at the site of infection (i.e., lung) coupled to a failure to generate a local and incremental IFN-γ response^35^; (ii) In a steroid only based model of immuno-suppression in BALB/c mice that were given IFN-γ replacement therapy, it was the specific administration of exogenous IFN-γ that decreased mortality.^36^ This has now been confirmed in patients with invasive fungal infections.^8,9,37^Taken together, these observations have established the importance of IFN-γ in invasive aspergillosis.

In healthy mice, there is a primary phase of rapid and intense clearance of *A. fumigatus* by both resident alveolar macrophages and recruited neutrophils by the innate immune system. This phase is associated with increased TNF-α.^18^ A second phase then follows during which fungal debris is removed by incoming monocytes. TNF-α returns to normal and healing of the lung occurs. However, in steroid immuno-suppressed mice with invasive aspergillosis, it has become clear that although TNF-α still plays an early and crucial role, the clearance of spores by alveolar macrophages is altered by a delay in the production of TNF-α.^18^ The outcome is invasion of lung by hyphae, recruitment of numerous neutrophils, and widespread tissue necrosis.^38^

Our animal model studies are based upon the use of aerosolised AP as pre-exposure prophylaxis. In a study by Schmitt et al of invasive aspergillosis in hydrocortisone immuno-suppressed rats that were given aerosolised deoxycholate-AmB (Fungizone), a single prophylactic dose of 1.6 mg/kg AmB was given 2 days before infection.^27^ The result was 80% survival of mice at day + 8. Using their method of AmB dose calculation, our daily inhaled dose of AmB was 135 μg/kg making a total 3 day dose of 405 μg/kg; this total dose of AmB was 75% less than the aerosolised dose of deoxycholate-AmB (Fungizone) reported by Schmitt.^27^ Furthermore, using aerosolised AP rather than deoxycholate-AmB (Fungizone) avoids the cytotoxic and lytic effects of deoxycholic acid in the lung,^6^ as discussed below. AP also offers the additional advantage of a slow, pH mediated, and tissue targeted release of the active AmB in high concentration at the intracellular site where fungal pathogens initiate infection, survive and persist. Taken together, this means greater drug efficacy at the actual site of infection at a lower dose administered.

Remarkably, we achieved 100% survival at day + 8 with the pre-exposure prophylaxis regimen described. However, the < 1% of persisting *Aspergillus* spores in alveolar macrophages (Figure 3, *E* and *F*) showed some evidence of spore germination (Figure 7). Alveolar macrophages in normal healthy mice can rapidly clear germinated as well as ungerminated *A. fumigatus* spores. However, in steroid immuno-suppressed mice, some spores can start germinating within 2-4 h of inhalation because they are not killed by alveolar macrophages^39,40^; ungerminated spores have been found in the lungs of those mice that survive infection. This is consistent with our findings at day + 2 (Figure 3, *D*-*F*) in mice given pre-exposure prophylaxis with AP in that spores, but not hyphae, were occasionally seen. It suggests that an effective immune response is only generated against the hyphae of *A. fumigatus* and that persisting non-germinating spores do not, of themselves, initiate a host immune response in the lung.^41^ In this specific context, it is important to note that AmB has no anti-fungal activity against spores.^42,43^ This means that the lungs cannot be completely sterilised of spores with AmB, or by any other antifungal drug, and that some post-exposure prophylaxis with AP is likely to be required to kill any persisting spores if and when they start to germinate.

Several studies have reported on the use of aerosolised AmB as both the deoxycholate and lipid formulations in rodents and humans.^44-47^ Important factors for effective nebulisation of AmB based drugs are particle size (diameter 2-5 μm) and use of an isotonic solution in the nebuliser. Current clinical protocols recommend that aerosolised AmB should be given three times a day in a dose of 200 μg/kg every 8 h for the first month post-transplant followed by a once daily dose of 500 μg/kg/day for up to 6 months post-transplant.^48^ A worldwide survey of antifungal prophylaxis in lung transplantation found that 58.6% of centres were employing antifungal prophylaxis with 97.1% specifically targeting *Aspergillus* species. Oral voriconazole alone or in combination with inhaled AmB were the preferred first line agents in current use. Six months post lung transplantation, 51.8% of centres no longer used antifungal prophylaxis.^49^

The major side effects of the deoxycholate-AmB in current clinical use are cough and bronchospasm.^50^ This is due to the damage caused to the lung epithelial surface by the dose dependent inhibition of surfactant function by deoxycholic acid.^51^ Although liposomal AmB is better tolerated,^52^ there are recent concerns about its use with particular reference to the lung because it prevents IFITM3-mediated restriction of Influenza A virus replication. This removes > 60% of the protective effects of interferons against Influenza A virus.^53^

In conclusion, PMA polymers and hydrogel capsules are in increasingly widespread pharmaceutical use as a mature biomaterial platform. Mixtures of oppositely charged polyelectrolytes can easily be made in water to form discrete and stable nanoparticles with a polyanionic coat. This enables insoluble and/or toxic drugs, like AmB, to be made into safe water soluble nanoparticles. We have made an AP nanoparticle that is based upon the use of very small 3.52 kDa PMA and AmB and have shown that is not toxic to human or mouse cells of lung epithelium and macrophage origin in both healthy mouse lung and in *Aspergillus* infected lung. This study therefore provides *in-vivo* proof of concept that very small and cost-effective nanoparticles can be simply designed and developed for use as pre-exposure prophylaxis agents to effectively deliver anti-microbial drugs to the lung.

### Ethics statement

Animal studies were performed under UK licence PPL 70/7324.

## Appendix A Supplementary data

The following in the supplementary data to this article.

## References

[1] Yessine M.-A., Leroux J.-C. (2004). Membrane-destabilizing polyanions: interaction with lipid bilayers and endosomal escape of biomacromolecules. Adv Drug Deliv Rev.

[2] Obeidat W.M., Abuznait A.H., Sallam A.S. (2010). Sustained release tablets containing soluble polymethacrylates: comparison with tableted polymethacrylate IPEC polymers. AAPS PharmSciTech.

[3] Zelikin A.N., Price A.D., Stadler B. (2010). Polymethacrylic acid polymer hydrogel capsules: drug carriers, sub-compartmentalized microreactors, artificial organelles. Small.

[4] Maher S., Leonard T.W., Jacobsen J., Brayden D.J. (2009). Self-assembled hydrogels are also being developed that can encapsulate hydrophobic drugs for sustained drug delivery. Adv Drug Deliv Rev.

[5] Gajanayake T., Olariu R., Leclère F.M., Dhayani A., Yang Z., Bongoni A.K. (2014). A single localized dose of enzyme-responsive hydrogel improves long-term survival of a vascularized composite allograft. Sci Transl Med.

[6] Corware K., Harris D., Teo I., Rogers M., Naresh K., Muller I. (2011). Accelerated healing of cutaneous leishmaniasis in non-healing BALB/c mice using water soluble amphotericin B-polymethacrylic acid. Biomaterials.

[7] Nakamura K., Maitani Y., Lowman A.M., Takayama K., Peppas N.A., Nagai T. (1999). Uptake and release of budesonide from mucoadhesive pH-sensitive copolymers and their application to nasal delivery. J Control Release.

[8] Armstrong-James D., Teo I.A., Shrivastava S., Petrou M.A., Taube D., Dorling A. (2010). Rapidly curative exogenous interferon-γ immuno-therapy for invasive fungal infections in renal transplant patients. Am J Transplant.

[9] Armstrong-James D., Teo I.A., Herbst S., Petrou M., Shiu K.Y., McLean A. (2012). Renal allograft recipients fail to increase interferon-γ during invasive fungal diseases. Am J Transplant.

[10] Denning D.W. (1988). Invasive aspergillosis. Clin Infect Dis.

[11] Latge J.P. (1999). *Aspergillus fumigatus* and aspergillosis. Clin Microbiol Rev.

[12] Baddley J.W., Andes D.R., Marr K.A., Kontoyiannis D.P., Alexander B.D., Kauffman C.A. (2010). Factors associated with mortality in transplant patients with invasive aspergillosis. Clin Infect Dis.

[13] Pasqualotto A. (2009). Differences in pathogenicity and clinical syndromes due to *Aspergillus fumigatus* and *Aspergillus flavus*. Med Mycol.

[14] Pfaller M.A., Boyken L., Hollis R.J., Messer S.A., Tendolkar S., Diekema D.J. (2006). Global surveillance of in vitro activity of micafungin against *Candida*: a comparison with caspofungin by CLSI-recommended methods. J Clin Microbiol.

[15] White P.L., Linton C.J., Perry M.D., Johnson E.M., Barnes R.A. (2006). The evolution and evaluation of a whole blood PCR assay for the detection of invasive aspergillosis in haematology patients in a routine clinical setting. Clin Infect Dis.

[16] Teo I., Toms S.M., Marteyn B., Barata T.S., Simpson P., Johnston K.A. (2012). Preventing acute gut wall damage in infectious diarrhoeas with glycosylated dendrimers. EMBO Mol Med.

[17] Herbst S., Shah A., Carby M., Chusney G., Kikkeri N., Dorling A. (2013). A new and clinically relevant murine model of solid-organ transplant aspergillosis. Dis Model Mech.

[18] Herbst S., Shah A., Moya M.M., Jensen B., Reed A., Birrel M. (2015). Phagocytosis-dependent activation of the TLR9-BTK-calcineurin-NFAT pathway co-ordinates innate immunity to *Aspergillus fumigatus*. EMBO Mol Med.

[19] Manunta M.D.I., McAnulty R.J., Tagalakis A.D., Bottoms S.E., Campbell F., Hailes H.C. (2011). Nebulisation of receptor-targeted nanocomplexes for gene delivery to the airway epithelium. PLoS One.

[20] Graybill J.R., Bocanegra R., Najvar L.K., Loebenberg D., Luther M.F. (1998). Granulocyte colony-stimulating factor and azole antifungal therapy in murine aspergillosis: role of immune suppression. Antimicrob Agents Chemother.

[21] Les K.A., Mohamed-Ahmed A.H.A., Balan S., Choi J.-W., Martin D., Yardley V. (2014). Poly(methacrylic acid) complexation of amphotericin B to treat neglected diseases. Polym Chem.

[22] Merkow L.P., Epstein S.M., Sidransky H., Verney E., Pardo M. (1971). An ultrastructural study of alveolar macrophages after phagocytosis of *A. flavus* spores in vivo. Am J Pathol.

[23] Lass-Flörl C. (2012). *Aspergillus terreus*: how inoculum size and host characteristics affect its virulence. J Infect Dis.

[24] Mandel M., Leyte J.C., Stadhouder M.G. (1967). The conformational transition of polymethacrylic acid in solution. J Phys Chem.

[25] Ikawa T., Abe K., Honda K., Tsuchida E. (1975). Interpolymer complex between polyethylene oxide and polycarboxylic acid. J Polym Sci.

[26] Khutoryanskiy V.V. (2007). Hydrogen bonded interpolymer complexes as materials for pharmaceutical applications. Int J Pharm.

[27] Schmitt H.J., Bernard E.M., Hauser M., Armstrong D. (1988). Aerosol amphotericin B is effective for prophylaxis and therapy in a rat model of pulmonary aspergillosis. Antimicrob Agents Chemother.

[28] Schaffner A., Douglas H., Braude A.I., Davis C.E. (1983). Killing of *Aspergillus* spores depends on the anatomical source of the macrophage. Infect Immun.

[29] Merigan T.C., Finkelstein M.S. (1968). Interferon stimulating and in vivo antiviral effects of various synthetic anionic polymers. Virology.

[30] Otterlei M., Ostgaard K., Skjåk-Braek G., Smidsrød O., Soon-Shiong P., Espevik T. (1991). Induction of cytokine production from human monocytes stimulated with alginate. J Immunother.

[31] Flo T.H., Ryan L., Latz E., Takeuchi O., Monks B.G., Lien E. (2002). Involvement of TLR2 and TLR4 in cell activation by mannuronic acid polymers. J Biol Chem.

[32] Hu X., Li W.-P., Meng C., Ivashkiv L.B. (2003). Inhibition of IFN-γ signaling by glucocorticoids. J Immunol.

[33] Sallowski C., Kopydlowski K., Blanco J., Cody M.J., McNally R., Vogel S.N. (1999). IL-12 is dysregulated in macrophages from IRF-1 and IRF-2 knockout mice. J Immunol.

[34] Vila-del Sol V., Punzón C., Fresno M. (2008). IFN-γ induced TNF-α expression is regulated by interferon regulatory factors 1 and 8 in mouse macrophages. J Immunol.

[35] Armstrong-James D.P.H., Turnbull S.A., Teo I., Stark J., Rogers N.J., Rogers T.R.F. (2009). Impaired interferon-γ responses, increased interleukin-17 expression, and a TNF-α transcriptional program in invasive aspergillosis. J Infect Dis.

[36] Nagai H., Guo J., Choi H., Kurup V. (1995). Interferon-γ and TNF-α protect mice from invasive aspergillosis. J Infect Dis.

[37] Delsing C.E., Gresnigt M.S., Leentjens J., Preijers F., Frager F.A., Kox K. (2014). Interferon-γ as adjunctive immuno-therapy for invasive fungal infections: a case series. BMC Infect Dis.

[38] Duong M., Ouellet N., Simard M., Bergeron Y., Olivier M., Bergeron M.G. (1998). Kinetic study of host defense and inflammatory response to *Aspergillus fumigatus* in steroid induced immunosuppressed mice. J Infect Dis.

[39] White L.O. (1977). Germination of *Aspergillus fumigatus* conidia in the lungs of normal cortisone-treated mice. Sabouraudia.

[40] Waldorf A.R., Levitz S.M., Diamond R.D. (1984). In-vivo bronchoalveolar macrophage defense against *Rhizopus oryzae* and *Aspergillus fumigatus*. J Infect Dis.

[41] Slesiona S., Ibrahim-Granet I., Olias P., Brock M., Jacobsen I.D. (2012). Murine infection models for *Aspergillus terreus* pulmonary aspergillosis reveal long-term persistence of conidia and liver degeneration. J Infect Dis.

[42] Vitale R.G., Mouton J.W., Afeltra J., Meis J.F.G.M., Verweij P.E. (2002). Method for measuring postantifungal effect in *Aspergillus* species. Antimicrob Agents Chemother.

[43] Vitale R.G., Meis J.F.G.M., Mouton J.W., Verwij P.E. (2003). Evaluation of the post-antifungal effect of amphotericin B and nystatin against 30 zygomycetes using two different media. J Antimicrob Chemother.

[44] Allen S.D., Sorensen K.N., Nejdl M.J., Durrant C., Proffit R.T. (1994). Prophylactic efficacy of aerosolized liposomal (AmBisome) and non-liposomal (Fungizone) amphotericin B in murine pulmonary aspergillosis. J Antimicrob Chemother.

[45] Corcoran T.W., Venkataramanan R., Mihelc K.M., Marcinkowski A.L., Ou J., McCook B.M. (2006). Aerosol deposition of lipid complex amphotericin-B (Abelcet) in lung transplant recipients. Am J Transplant.

[46] Kuiper L., Ruijgrok E.J. (2009). A review on the clinical use of inhaled amphotericin B. J Aerosol Med Pulm Drug Deliv.

[47] Mead L., Danziger-Isakov L.A., Michaels M.G., Goldfarb S., Glanville A.R., Benden C. (2014). Antifungal prophylaxis in pediatric lung transplantation: an international multicentre survey. Pediatr Transplant.

[48] Solé A. (2008). Invasive fungal infections in lung transplantation: role of aerosolised amphotericin B. Int J Antimicrob Agents.

[49] Neoh C.F., Snell G.I., Kotsimbos T., Levvey B., Morrissey C.O., Slavin M.A. (2001). Antifungal prophylaxis in lung transplantation: a world-wide survey. Am J Transplant.

[50] Chishimba L., Langridge P., Powell G., Niven R.M., Denning D.W. (2014). Efficacy and safety of nebulised amphotericin B in severe asthma with fungal sensitization and allergic bronchopulmonary aspergillosis. J Asthma.

[51] Ruijgrok E.J., Vulto A.G., Van Etten Els W.M.N. (2001). Efficacy of aerosolized amphotericin B deoxycholate and liposomal amphotericin B in the treatment of invasive pulmonary aspergillosis in severely immunocompromised rats. J Antimicrob Chemother.

[52] Steinbach W.J., Benjamin D.K., Kontoyiannis D.P., Perfect J.R., Lutsar I., Marr K.A. (2010). Infections due to *Aspergillus terreus*: a multicenter retrospective analysis of 83 cases. Clin Infect Dis.

[53] Lin T.-Y., Chin C.R., Everitt A.R., Clare S., Perreira J.M., Savidis G. (2013). Amphotericin B increased influenza A virus infection by preventing IFITM3-mediated restriction. Cell Rep.

