# Supplementary material for: Nebulised amphotericin B-polymethacrylic acid nanoparticle prophylaxis prevents invasive aspergillosis
Source: Nanomedicine. 2015 Jul;11(5):1217–26. doi: 10.1016/j.nano.2015.02.012 (PMC4503863; doi:10.1016/j.nano.2015.02.012)
Supplement: Supplementary file 1 — Supplementary material. [file mmc1.pdf]

Supplementary Figure 1:

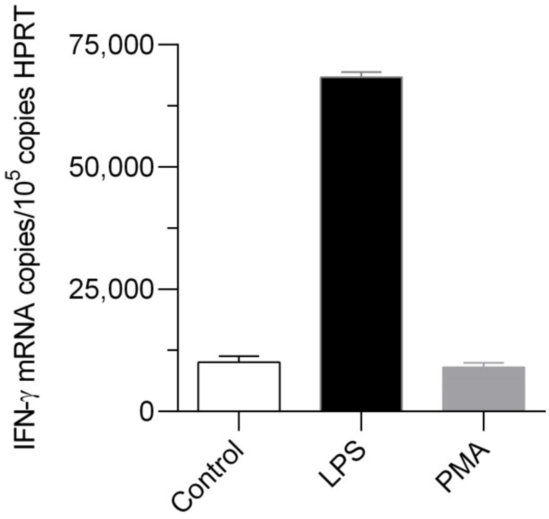

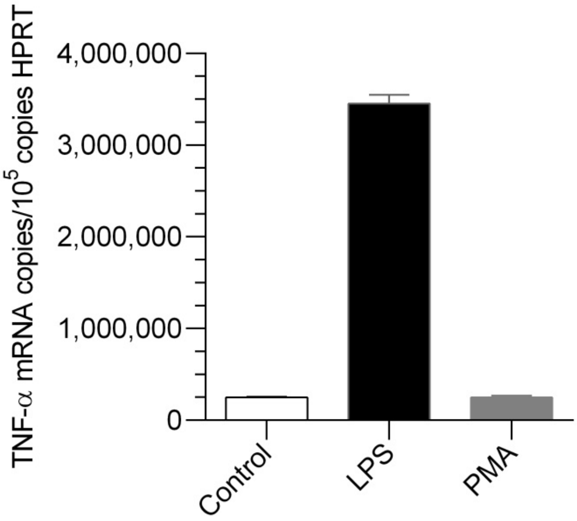

Lack of a pro-inflammatory effect of PMA-Na on monocyte derived macrophages:

PMA (3.52 kDa) was incubated with monocyte-derived-macrophages for 3 h at 25 µg/ml. It did not stimulate IFN-γ or TNF-α (n = 9). Similar results were obtained with 1.27 kDa, 1.7 kDa, 7.75 kDa and 18.5 kDa PMA.

Supplementary Figure 2:

UV spectra of AmB-PMA (3.52 kDa) and deoxycholate-AmB (Fungizone, Squibb).
The pale yellow powders were reconstituted in water at a final concentration of 10 µg/ml AmB.

AmB-PMA (3.52 kDa)

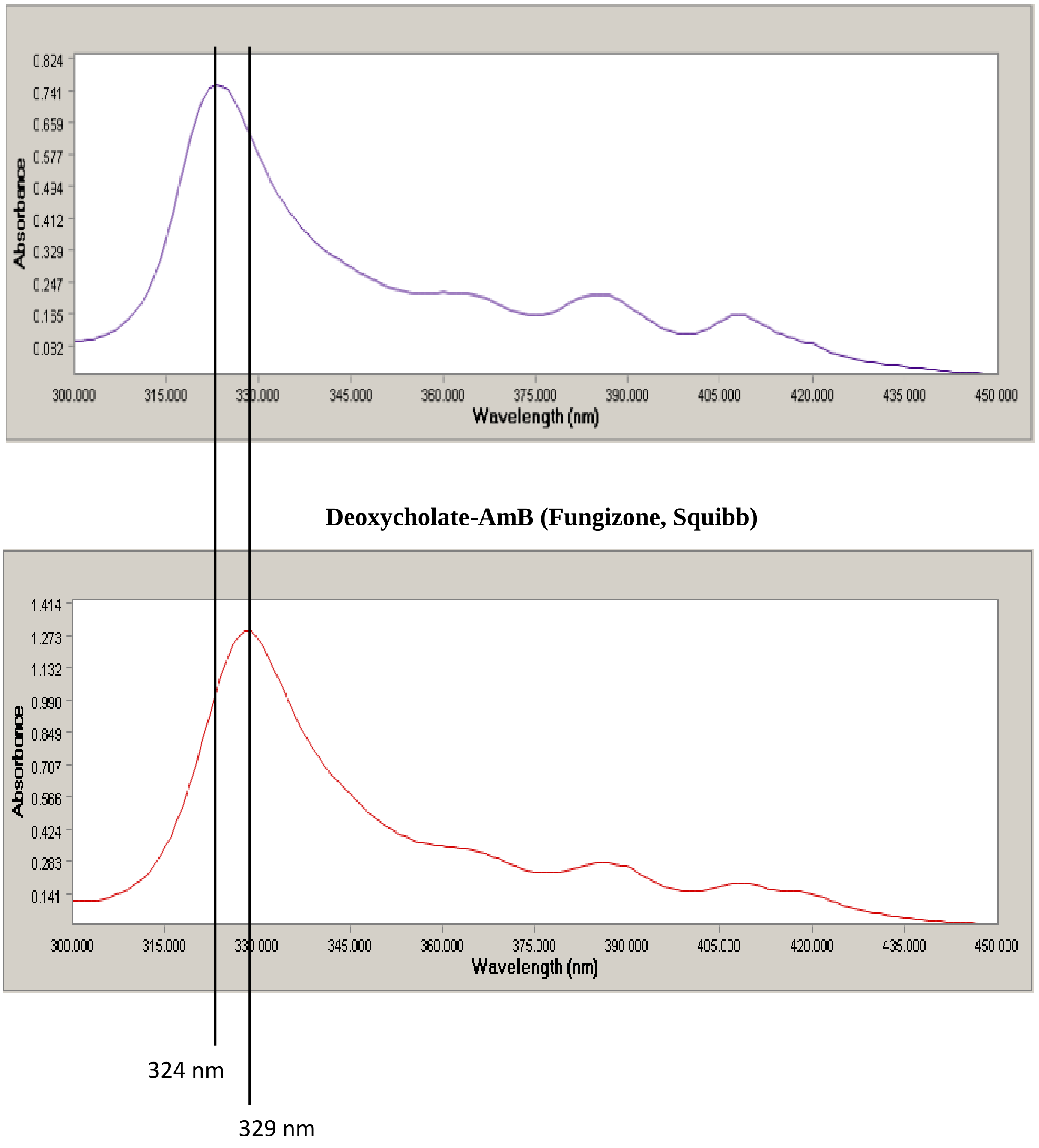

The AmB is highly aggregated in both deoxycholate-AmB (Fungizone, Squibb) and in liposomal AmB (Ambisome, Gilead). The λ_{max} of the UV spectra of deoxycholate-AmB was 329 nm (shown in red) and of liposomal AmB was 324 nm (not shown; see Reference 6). The λ_{max} of the UV spectra of AmB-PMA (3.52 kDa) (AP) was 324 nm (shown in blue). Similar results were obtained with 1.27 kDa, 1.7 kDa, 7.75 kDa and 18.5 kDa PMA.

Supplementary Figure 3:

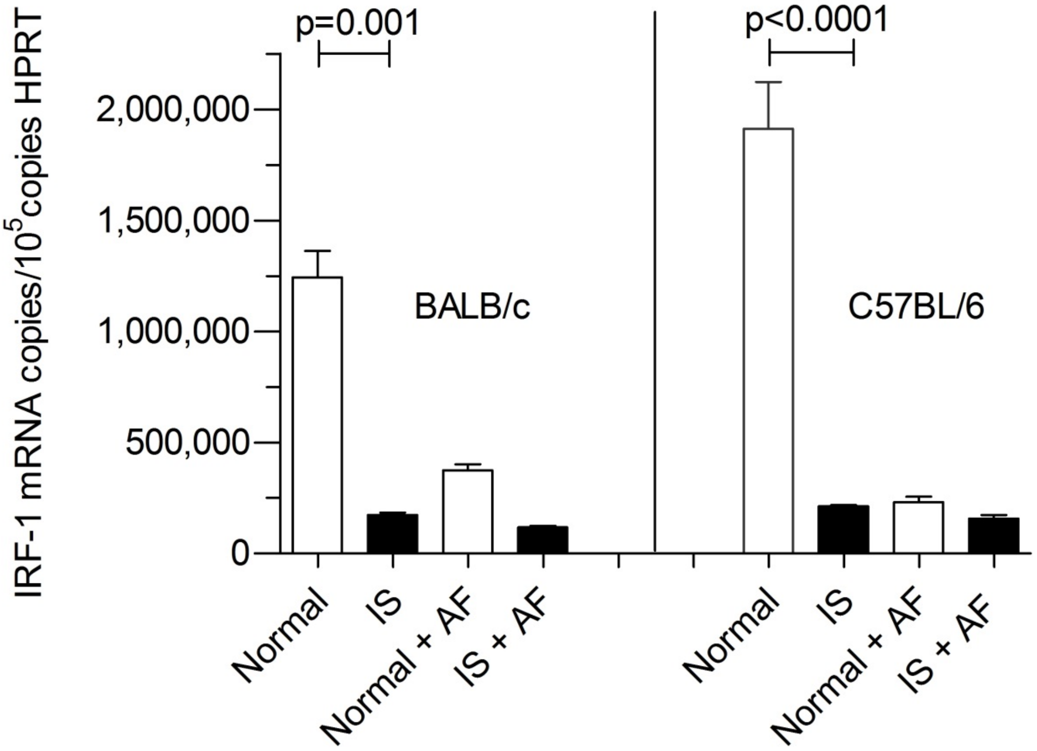

Comparison of IRF-1 in BALB/c and C57BL/6 mouse lung at day +2:

Immuno-suppression caused a large and significant suppression of Interferon Regulatory Factor (IRF) -1 that did not change after infection, or with AF pre-exposure prophylaxis (n = 4).

For BALB/c mice:
Normal IRF-1 = 1.24 $x10^6$ ± 118,500 copies/10^5 copies HPRT; IS + AF IRF-1 = 118,000 ± 7,890 copies/10^5 copies HPRT.

For C57BL/6 mice:
Normal IRF-1 = 1.91 $x10^6$ ± 210,800 copies/10^5 copies HPRT; IS + AF IRF-1 = 157,300 ± 16,200 copies/10^5 copies HPRT.

Supplementary Table 1:

Isolate	Name	**AmB-PMA (1.27 kDa)**	**AmB-PMA (1.7 kDa)**	**AmB-PMA (3.52 kDa)**	**AmB-PMA (7.75 kDa)**	**AmB-PMA (18.5 kDa)**	**Fungizone**
		MIC (µg/ml)	MIC (µg/ml)	MIC (µg/ml)	MIC (µg/ml)	MIC (µg/ml)	MIC (µg/ml)
J1	*A. fumigatus*	0.25	0.25	0.125	0.125	0.5	0.125
J2	*A. fumigatus*	0.25	0.25	0.125	0.125	0.25-0.5	0.25
J3	*A. fumigatus*	0.125	0.125	0.0625	0.0625	0.25	0.0625
J4	*A. fumigatus*	0.25	1.0	0.125	0.125	0.5	0.125-0.25
J5	*A. fumigatus*	0.25	1.0	0.125	0.125	0.5	0.125-0.25
HH-7	*A. fumigatus*	1.0	0.25	0.5	0.5	2.0	0.5
HH-12	*A. fumigatus*	0.5	0.25	0.25	0.25	1.0	0.25-0.5
HH-23	*A. fumigatus*	0.25	0.5	0.125	0.125	0.5	0.125-0.25
HH-24	*A. fumigatus*	0.5	0.5	0.25	0.25	1.0	0.25
HH-55	*A. fumigatus*	0.25	0.0625	0.125	0.125	0.25	0.125
CEA10	*A. fumigatus*	0.0625	0.25	0.0312	0.0312	0.125	0.0312

Summary of the MIC test results shown above:

**Nanoparticle**	**Range of MICs (µg/ml)**	**Mean of MICs (µg/ml)**
AmB-PMA (1.27 kDa)	0.0625 - 1	0.25
AmB-PMA (1.7 kDa)	0.0625 - 1	0.25
AmB-PMA (3.52 kDa)	0.0312 - 0.5	0.125
AmB-PMA (7.75 kDa)	0.0312 - 0.5	0.125
AmB-PMA (18.5 kDa)	0.125 - 2	0.5 - 1.0
Fungizone	0.0312 - 0.5	0.25

The antifungal activity of the different AmB-PMAs was determined against 11 isolates of *A. fumigatus* up to a final concentration of 16 µg/ml. AmB-PMA (1.27 kDa & 1.7 kDa) had the same MIC as Fungizone (0.25 µg/ml). In contrast, AmB-PMA (3.52 & 7.75 kDa) had better antifungal activity at 0.125 µg/ml when compared to Fungizone. The anti-fungal activity of the AmB-PMA (18.5 kDa) was 0.5 - 1 µg/ml.
